# Thickness dependence of the interfacial Dzyaloshinskii–Moriya interaction in inversion symmetry broken systems

**DOI:** 10.1038/ncomms8635

**Published:** 2015-07-08

**Authors:** Jaehun Cho, Nam-Hui Kim, Sukmock Lee, June-Seo Kim, Reinoud Lavrijsen, Aurelie Solignac, Yuxiang Yin, Dong-Soo Han, Niels J. J. van Hoof, Henk J. M. Swagten, Bert Koopmans, Chun-Yeol You

**Affiliations:** 1 Department of Physics, Inha University, Incheon 402-751, Republic of Korea.; 2 Department of Applied Physics, Center for NanoMaterials, Eindhoven University of Technology, PO Box 513, Eindhoven 5600 MB, The Netherlands.

## Abstract

In magnetic multilayer systems, a large spin-orbit coupling at the interface between heavy metals and ferromagnets can lead to intriguing phenomena such as the perpendicular magnetic anisotropy, the spin Hall effect, the Rashba effect, and especially the interfacial Dzyaloshinskii–Moriya (IDM) interaction. This interfacial nature of the IDM interaction has been recently revisited because of its scientific and technological potential. Here we demonstrate an experimental technique to straightforwardly observe the IDM interaction, namely Brillouin light scattering. The non-reciprocal spin wave dispersions, systematically measured by Brillouin light scattering, allow not only the determination of the IDM energy densities beyond the regime of perpendicular magnetization but also the revelation of the inverse proportionality with the thickness of the magnetic layer, which is a clear signature of the interfacial nature. Altogether, our experimental and theoretical approaches involving double time Green's function methods open up possibilities for exploring magnetic hybrid structures for engineering the IDM interaction.

## Abstract (web summary)

Spin-orbit effects at heavy metal/ferromagnet interfaces can give rise to the Dzyaloshinskii-Moriya interaction, promoting chiral magnetization textures applicable in thin film devices. Here, the authors use Brillouin light scattering to study the dependence of this interaction on film thickness.

## Graphical abstract

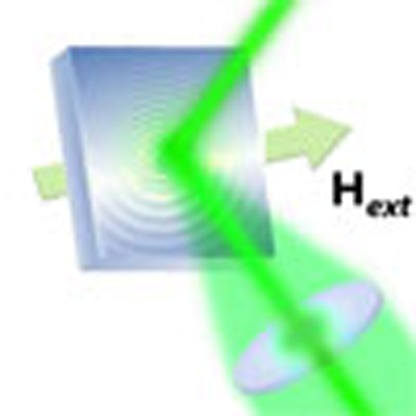

In the presence of spin-orbit coupling at interfaces on low-dimensional magnetic heterojunction structures, the effect of structural inversion asymmetry leads to an additional anisotropic exchange term, namely the interfacial Dzyaloshinskii–Moriya (IDM) interaction^1^,^2^,^3^,^4^ (already predicted by Fert in 1980), which is a branch of the Dzyaloshinskii–Moriya (DM) interaction^5^,^6^. This interfacial phenomenon has been recently re-illuminated and experimentally demonstrated because of its massive potentials to explore new magnetic behaviours such as chiral domain wall (DW) dynamics^7^,^8^,^9^,^10^,^11^,^12^,^13^ and skyrmions^14^,^15^,^16^. To develop this field of DW devices and skyrmionics (the latter with great promises for superior nanoelectronics devices), experimental tools to extract the magnitude and sign of IDM interaction are urgently required. However, contrary to bulk-type DM interaction measurements^17^, recent extensive experiments clearly observed the existence of the IDM interaction, but magnetic field and electric current driven DW dynamics measurements were definitely linked to the perpendicular magnetic anisotropy (PMA)^8^,^9^,^10^,^11^,^12^,^13^. At present, to further explore independent by the underlying physics of the IDM interaction without any other linked material parameters, a radically different experimental approach is strongly required.

In this article we measure the ferromagnetic layer thickness dependent of IDM interaction quantitatively and qualitatively. Inelastic light scattering, so-called Brillouin light scattering (BLS), is performed to observe non-reciprocal spin wave (SW) dispersion relations affected by the IDM interaction^18^. The detailed explanation about the BLS is shown in Supplementary Note 1. The advantages of BLS to determine the IDM energy density is described in Supplementary Note 2. Our main findings are twofold: first, the inverse proportionality of the IDM energy densities to the ferromagnetic layer thickness shows that the IDM interaction is purely originated from the interfaces, and second, we present a state-of-the-art quantum-mechanical approach to confirm the asymmetric dispersion relations and the inverse proportionality of the IDM interaction. As representative heterostructures, Pt/Co/AlO_x_ and Pt/CoFeB/AlO_x_ are chosen because these multilayer structures are already predicted to have a large IDM interaction^12^.

## Results

### SW Frequency differences due to the IDM interaction

**Figure 1:**
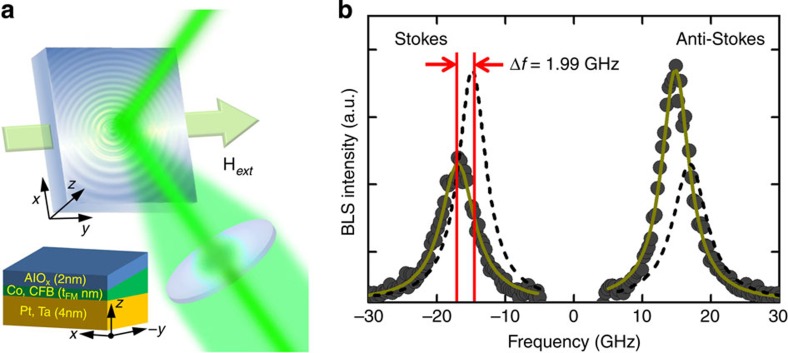
The BLS measurements. (**a**) Schematic configuration of the BLS measurement. The external magnetic field is applied along the *y*-direction and a *p*-polarized laser beam excites two interfaces SWs along the *x*-direction. Inset: schematic picture of wedge-type sample geometry. (**b**) The BLS spectrum with a magnetic field *H*_ext_=0.69 T. The incident angle is fixed at *θ*=45° (*k*_*x*_=0.0167, nm^−1^). To identify the frequency differences (Δ*f*) between Stokes (negative frequency region) and anti-Stokes (positive frequency region), mirrored curves are drawn as black dashed line. The red vertical lines indicate the centre of the SW frequency and red arrows indicate the Δ*f*, here 1.99 GHz. The black circles refer to the experimental result and dark yellow solid line is the Lorentzian fitting curve. The data accumulation time for each spectrum is about 60 min.

Propagating SWs on a magnetic thin film can be localized at the top and bottom surfaces of the ferromagnetic layer when the wavevector *k* of the SW is perpendicular to the magnetization of the system. This SW mode is namely Damon–Eshbach (DE) mode (often called surface mode)^19^ and it is indeed one of the appropriate physical quantities to investigate the interface effect, especially affected by the IDM interaction. To realize the DE geometry, we first apply an external magnetic field along the in-plane as depicted in Fig. 1a. Simply, BLS measures the scattered light from two interfaces, which contains photons at frequencies shifted by the frequencies of excited SWs. In this inelastic process, the photon loses its kinetic energy (Stokes process) to create one of quasi-particles (SW in our study) or gains energy (anti-Stokes process) by absorbing one. Consequently, these spectral components can determine the frequencies and intensities of SWs existing at the point in the sample where the incident light is focused (see Methods).

Usually, the SW frequencies of Stokes and anti-Stokes peaks should be at the same position or slightly different due to the PMA energy difference between top and bottom interfaces of the ferromagnet^20^. However, recent theoretical and numerical calculation proposes a prominent clue that the frequencies and the attenuation lengths of the travelling SWs with opposite wavevectors (±*k*) are significantly different due to the IDM interaction, and then these characteristics of the SWs are measurable^21^. For BLS, the frequency difference (Δ*f*) indicates the mismatch between the frequencies of Stokes and anti-Stokes peaks. We report that a large frequency difference (Δ*f*=1.99 GHz) for Pt/Co(1.2 nm)/AlO_x_ is obviously observed as shown in Fig. 1b. Now, one crucial fact can be emphasized that the GHz range of the Δ*f* is a clear signature of the IDM interaction. The details will be further discussed later.

### Magnetic field dependence

**Figure 2:**
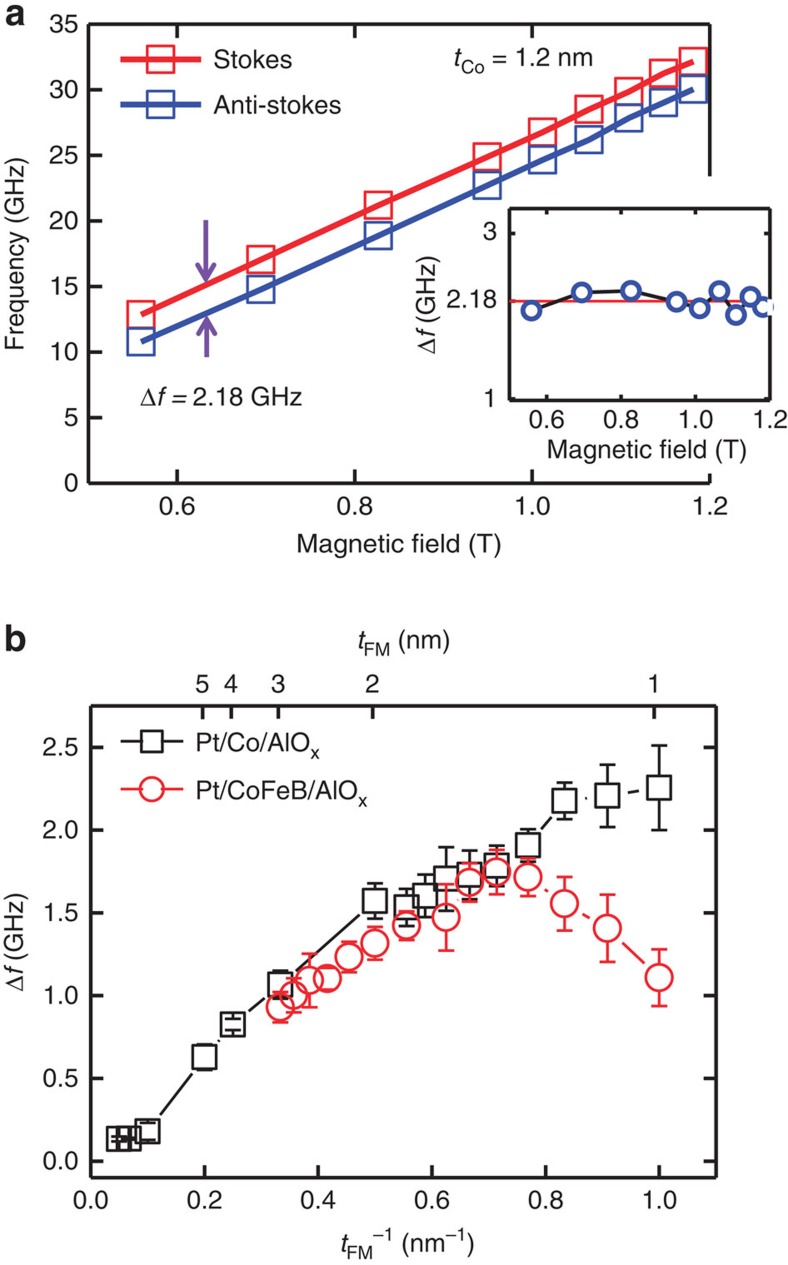
The *H*_ext_ dependence measurement and the Δ*f* between Stokes and anti-Stokes peak. (**a**) Magnetic field-dependent BLS measurements at *t*_Co_=1.2 nm. The in-plane magnetic field varies from 0.5 to 1.2 T and the angle of the incident light is fixed at *θ*=45°. The violet arrows are average Δ*f* is 2.18 GHz between Stokes (red squares and line) and anti-Stokes (blue squares and line) peaks. Inset: the frequency differences (Δ*f*) as a function of applied magnetic field. (**b**) Δ*f* as a function of *t*_FM_^−1^ for two different magnetic materials (Co and CoFeB). Black squares and red circles indicate Δ*f* for Co and CoFeB, respectively. For these measurements, the incident angle is fixed at *θ*=45°, which corresponds to the *k*_*x*_=0.0167, nm^−1^. Error bars correspond to the s.d. of the BLS measurements.

To precisely and systematically investigate this interface effect by means of BLS, two different methods (magnetic field dependence and incident angle dependence) as a function of the thickness of the ferromagnet (*t*_FM_) are proposed in this work. We now discuss the details of two different methods successively. The DE SW frequencies (dispersions) including the IDM interaction are given as^21^:


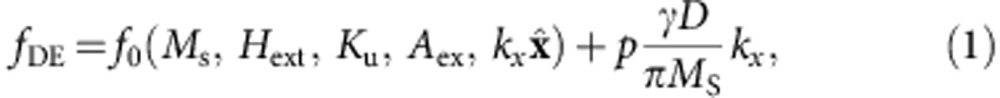


where *f*_0_ is the SW frequency without the IDM contribution, *H*_ext_, *K*_u_, *A*_ex_, *γ*, *p* and 
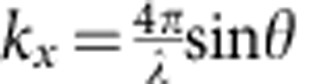
 are the external magnetic field, the magnetic anisotropy, the exchange stiffness, the gyromagnetic ratio, the polarity of the magnetic field (*p*=±1) and the wavevector of the SW, where *θ* is the incident angle of the light, respectively. Therefore, the definition of the frequency difference is simply derived as 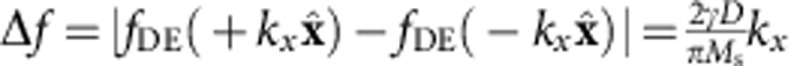, where *M*_s_ and *D* are the saturation magnetization and the IDM energy density, respectively. This SW dispersion apparently shows that the Δ*f* is invariant while the magnetic field increases (or decreases). So, the field-dependent measurement allows us to minimize the uncertainties of the measured Δ*f.* The measured SW frequencies of Stokes and anti-Stokes peaks as a function of the magnetic field for Pt/Co(1.2 nm)/AlO_x_ are shown in Fig. 2a. Two SW frequencies increase with increasing applied magnetic field, but the Δ*f* (the averaged Δ*f* is 2.18 GHz) is indeed a constant (see the inset in Fig. 2a). From these field-dependent measurements, we can convert to the IDM energy densities because the SW wavevector is fixed at *k*_*x*_=0.0167, nm^−1^ (*θ*=45°). It must be noted that the minimum applied in-plane field is 0.5 T is large enough to pull the magnetization in the plane. Therefore, the observed Δ*f* is only for the in-plane magnetization, and we cannot conclude that Δ*f* will vanish or not when the magnetization is out of plane. Due to the limitation of BLS measurement setup, it is hard to determine Δ*f* for the out-of-plane magnetization (see additional Supplementary Note 3 and Supplementary Fig. 1).

In many magnetic systems, interface effects can be identified by an inverse proportionality to the ferromagnetic layer thickness such PMA^22^, exchange bias^23^, switching current density of spin transfer torque^24^, the effective field of the interlayer exchange coupling^25^ and so on. In this point of view, we systematically measure Δ*f* as a function of the thickness of the ferromagnets (Co and CoFeB) and nine data points with different magnetic fields are averaged for each thickness. As shown in Fig. 2b, Δ*f* approaches to the origin when 
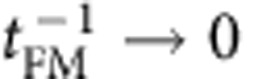
 for both Co and CoFeB samples by which we consequently confirm that the IDM interaction for our asymmetric structures is purely originated from the interface. For the thinner CoFeB cases (*t*_CoFeB_<1.6 nm), the frequency differences deviate from the inverse proportionality. It implies that the non-linear behaviour in Fig. 2b is due to the degradation of the interface quality (see Supplementary Note 4 and Supplementary Fig. 2).

### SW propagation direction dependence of Δ*f*

**Figure 3:**
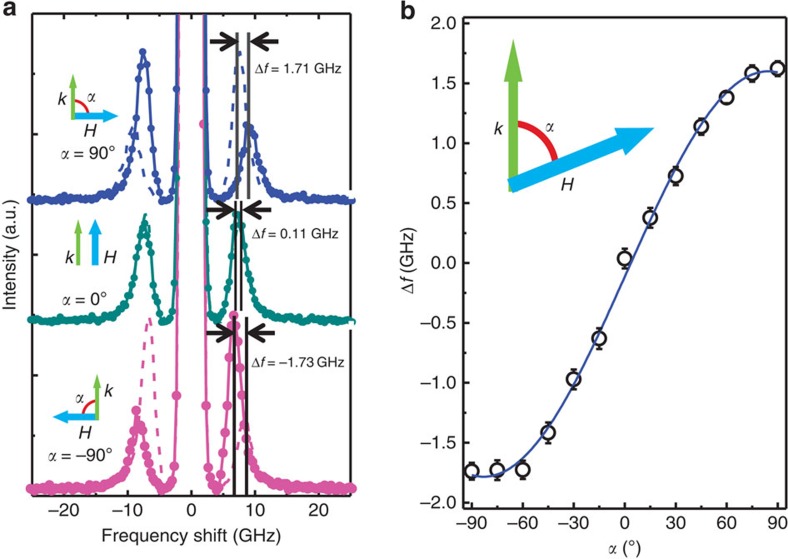
The BLS spectra for *α*=90° and 0°, and *α* dependence of Δ*f* for 2.0-nm-thick Co. (**a**) The frequency difference between Stokes and anti-Stokes are clearly observed (Δ*f*=+1.71, −1.73 GHz) for *α*=±90° (**k**_**||**_⊥**H**), while the Δ*f* (=0.11 GHz) for *α*=0° (**k**_**||**_//**H**) is less than BLS resolution. The black vertical lines and the black arrows indicate the Δ*f* between Stokes and anti-Stokes peaks. The green and blue arrows indicate the directions of the SW wavevector and the applied magnetic field, respectively. (**b**) The measured Δ*f* as a function of the *α*. The solid line is the fitting curve from equation (2). Error bars correspond to the s.d. of the BLS measurements.

We now demonstrate another proof that the asymmetric frequency differences indeed emerge from the interface. In recent previous theoretical work, Cortés-Ortuño^26^ claims that the frequency differences Δ*f* by the DM interaction can be expressed as:


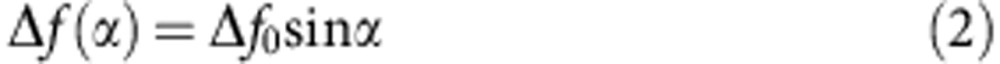


where *α* indicates the angle between **k**_||_ and **M**, and Δ*f*_0_ is a frequency difference at *α*=*π*/2. The physical interpretation of equation (2) is that the frequency differences Δ*f* is created by the energy differences of two propagating SWs for both interfaces. Since the IDM interaction introduces these energy differences, this equation is another clear evidence of the DM interaction, especially for the case of the interface effect. Figure 3 shows the angular dependence of the frequency differences between the angle of SW *k*-vector and the direction of **M**. Figure 3a indicates the case of *α*=±*π*/2 (usual BLS measurement geometry, that is, 
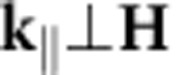
) and *α*=0° (90° rotation from usual BLS measurement geometry, that is, **k**_**||**_//**H**). It is clearly shown that Δ*f* (=+1.71, −1.73 GHz) are finite and opposite sign for *α*=±*π*/2, while Δ*f*=0.11 GHz for *α*=0° is less than BLS limitation (∼0.29 GHz, see the Supplementary Note 5 and Supplementary Fig. 3a,b). The systematic angular dependent measurements are shown in Fig. 3b and we overlap the sinusoidal curve from equation (2). As expected, they are in good agreement with each other.

### SW *k*-vector dependence

**Figure 4:**
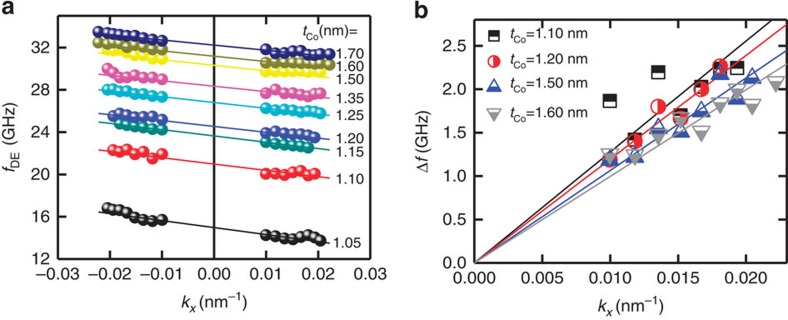
The SW dispersion relation and the linearity of the Δ*f* in each thickness. (**a**) The asymmetric dispersion relation measured by the BLS for various Co thicknesses. For these measurements, the applied magnetic field is fixed at *H*_ext_=0.915 T. The solid lines correspond to linear fit to the experimental results. For all Co thicknesses, the *f*_DE_ linearly decreases with increasing *k*_*x*_. These asymmetric and linear dispersion relations can be regarded as the direct evidence that the Δ*f* in our measurement is a consequence of IDM interaction. (**b**) All Δ*f* and linear fitting lines are visualized in one graph.

Furthermore, we measure the dispersion relation of SW (frequency versus wavevector) by varying the incident angle *θ* of the probing light, which determines the selected SW's wavevector 
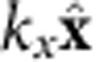
. We note that the magnetic field- and *k*_*x*_-dependent measurements span two different branches of equation (1), and those two independent measurements can provide more reliable results in the present study. The dependence of *f*_DE_ on *k*_*x*_ are plotted in Fig. 4a for various Co thicknesses. The solid lines correspond to linear fit to the experimental results. For all Co thicknesses, the *f*_DE_ linearly decreases with increasing *k*_*x*_. Following the equation (1), *f*_0_ and Δ*f* varies quadratically and linearly with *k*_*x*_, respectively. However, for the limited range of *k*_*x*_ (−0.03 nm^−1^<*k*_*x*_<+0.03 nm^−1^), the *f*_0_ are almost constant, accordingly, such significant variation in *f*_DE_ results from those in Δ*f*. Therefore, these asymmetric and linear dispersion relations can be regarded as the direct evidence that the Δ*f* in our measurement is a consequence of IDM interaction. Recently, the asymmetric SW dispersion relation has been experimentally observed by using spin-polarized electron energy loss spectroscopy in double layer Fe films^27^. For comparison, we also examined the dispersion relation for Pt(4 nm)/Co(0.6 nm)/Pt(4 nm) representing a symmetric structure, where the IDM interaction at the bottom and top interfaces of the FM are known to be approximately of the same magnitude but with the opposite sign, thus leading to zero IDM interaction. Interestingly, for the symmetric structure, no significant IDM interaction is observed (see Supplementary Note 5). Fig. 4b shows the Δ*f* versus |*k*_*x*_| for selected Co thickness, *t*_Co_=1.1, 1.2, 1.5 and 1.6 nm. For each film thickness, one obtains a clear linear dependence. From the slopes, we can extract IDM energy density using the relation of equation (1) together with the gyromagnetic ratio *γ* and the saturation magnetization *M*_s_ deduced from the further BLS measurements.

### The IDM energy densities

**Figure 5:**
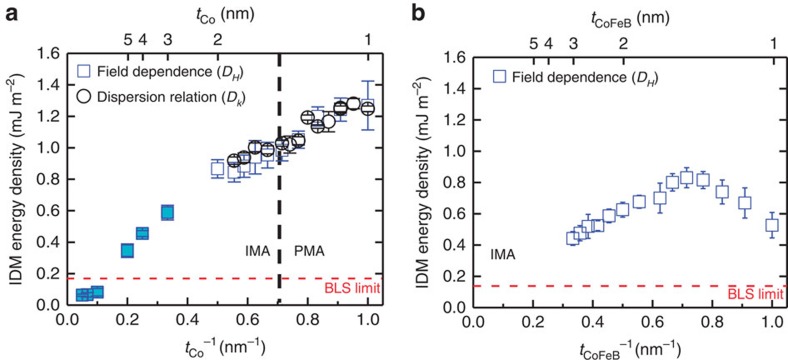
The IDM energy densities. (**a**) The IDM energy density as a function of *t*_Co_^−1^ for the two measurement methods. Blue squares and black circles show the IDM energy density measured by field dependence (*D*_*H*_) and dispersion relation (*D*_*k*_), respectively. The black dot line indicates the spin configuration changes PMA to in-plane magnetic anisotropy (IMA). (**b**) The IDM energy density as a function of *t*_CoFeB_^−1^ for the field dependence (*D*_*H*_). The red dot lines show the limit for our BLS setup. Error bars correspond to the s.d.

Next, we convert the measured Δ*f* to the IDM energy densities for our asymmetric structures as shown in Fig. 5. For Co samples, the measured IDM energy densities (*D*_*H*_ and *D*_*k*_ indicate the IDM energy densities from the field dependence and SW wavevector dependence, respectively) are in excellent agreement each other (see Fig. 5a). Figure 5b shows the measured IDM energy densities for the CoFeB sample. In this case, the effective magnetic anisotropy for all thicknesses is in-plane. The maximum IDM energy density is obtained to be about *D*=1.2 mJ m^−2^ (*D*=0.7 mJ m^−2^) for Pt/Co(1 nm)/AlO_x_ (Pt/CoFeB(1.6 nm)/AlO_x_). Recall that the saturation magnetization (*M*_s_) is one unique material parameter to convert the IDM energy density in equation (1). The saturation magnetization *M*_s_ (equal to 1,100 kA m^−1^ for Co and 948 kA m^−1^ for CoFeB) is determined by BLS measurement as well (see Supplementary Note 6 and Supplementary Fig. 4a,b).

### Numerically calculated SW dispersion relation

Two types of BLS measurements (magnetic field dependence and *k*_*x*_-vector dependence) are presented so far. From these measurements, we found the inverse proportionality of Δ*f*, which is a typical signature of the interfacial nature and the asymmetric dispersion relation. One of the main goals of the present work is to demonstrate the SW dispersion relation affected by the IDM interaction and the inverse proportionality not only by experiment but also theoretically. In previous work, theoretical evidences based on atomic-scale models^28^,^29^,^30^ have been reported. Udvardi *et al*.^28^ predict reciprocal SW dispersion relations for the specific crystallographic orientation in the Fe/W(110) by using first-principle calculations, without dipole–dipole interaction and external field, and Costa *et al*.^26^ provide dynamic susceptibilities (SW frequencies, life times and amplitudes) for ± SW vectors in the one or two monolayer (ML) of Fe on the W(110) based on multiband Hubbard model. Cortés-Ortuño and Landeros demonstrate reciprocal SW dispersion relations for different crystallographic classes. Here we introduce the numerical calculations for asymmetric SW dispersion relations and inverse proportionality by means of the double time Green's function technique, it is useful to study the thickness-dependent SW dispersion relations. This technique is well developed in statistical physics^30^ and magnetism^31^,^32^. The Hamiltonian with the IDM interaction for the finite thickness ferromagnetic layer in terms of the spin operators is given by^32^,^33^:


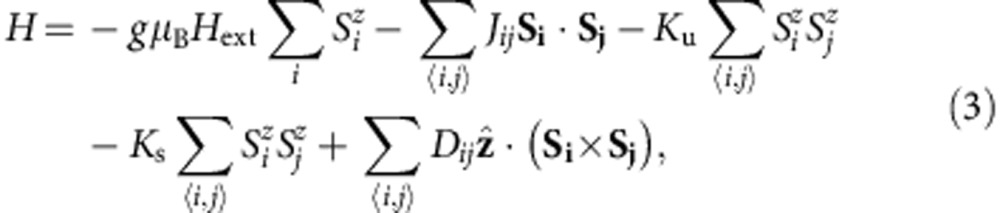


where *g* is the Landé *g*-factor, *J*_*ij*_ and *D*_*ij*_ are the isotropic inter-atomic Heisenberg and anisotropic DM exchange energies between the *i*-th and *j*-th spins, and *K*_u_ and *K*_s_ are the bulk and surface uniaxial anisotropy energies. In this model, we use different definition of coordinate system, we set the film normal along the *z*-axis. 〈*i*,*j*〉,〈*i*,*j*〉', and 〈*i*,*j*〉” denote the summations of the nearest neighbours. Here 〈*i*,*j*〉 is summation of all spins, 〈*i*,*j*〉' is for top and bottom interfaces, and 〈*i*,*j*〉” is only at the bottom interface where we assumed a heavy metal is placed. Therefore, we assume that the DM interaction exists only at the bottom interface. The SW dispersion relations can be obtained by solving equation (3). The detailed explanations and full derivations are shown in Supplementary Note 7 and Supplementary Fig. 5a,b.

**Figure 6:**
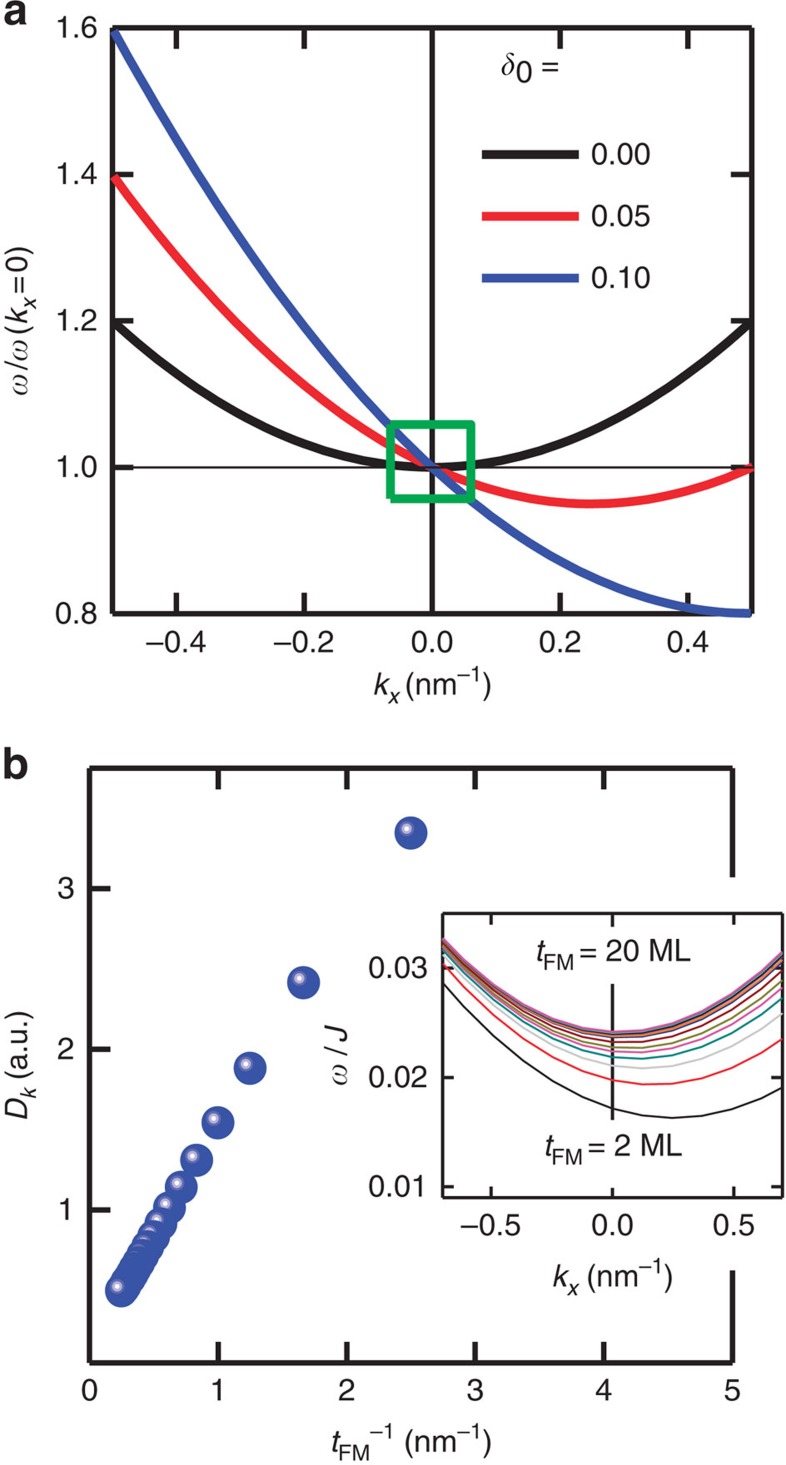
Numerically calculated SW dispersion relations. (**a**) Calculated SW dispersion relations for different IDM energy densities. The green box indicates that SW wavevector range of our BLS experiment. (**b**) The IDM energy density from the SW dispersion relations as a function of *t*_FM_^−1^. Inset: calculated SW dispersion relations for various *t*_FM_^−1^ from 2 ML to 20 ML when *δ*_0_ (*SD*/*J*_ex_*a*) is 0.1. The vertical line indicate the *k*_*x*_=0. The SW frequencies at *k*_*x*_=0 increase with increasing *t*_FM_.

Figure 6a shows numerically calculated SW dispersion relations for a ferromagnetic ML with the IDM interaction term *δ*_0_=*SD*/*J*_ex_*a*, where *S*, *a* and *J*_ex_ are spin number 1/2, the lattice constant and the exchange energy, respectively. A parabolic SW dispersion relation (black line) is obtained when *δ*_0_=0, when *δ*_0_ is non-zero, parabolic SW dispersion relations are shifted as given by equation (1) and shown by the red and blue lines for different strengths of *δ*_0_ ferromagnetic ML. As mentioned above, the SW 
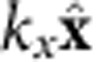
-vector of our BLS setup is limited from 0.0099 to 0.0205, nm^−1^; the small range is indicated by the green box in Fig. 6a. That is the reason that we obtained only linear behaviours of *f*_DE_ in Fig. 4a and one can be pointed out that this numerical result can sufficiently support our experimental data. Finally, the inverse proportionality of the IDM energy density as a function of the thickness of the ferromagnetic layer (*t*_FM_) is shown in Fig. 6b and the inset indicates asymmetric SW dispersion relations for *δ*_0_=0.1. In this calculation, we consider the thickness of the ferromagnetic layer from 2 ML to 20 ML. These full numerical SW dispersion relations reflect our experimental observations very well. First, the SW frequencies at *k*_*x*_=0 increase with increasing *t*_FM_. The experimental results show the same trend in Fig. 4a. Since the SW frequency is related with the interface PMA energy, it must be increased with increasing *t*_FM_ (see Supplementary Fig. 5a). Second, the parabolic SW dispersion relations have an additional linear *k*_*x*_. Because the coefficient of a linear *k*_*x*_ term is proportional to the *D*_*k*_, we can directly extract *D*_*k*_ from the SW dispersion relations. Very recently, there is another numerical and theoretical approach about the interface exchange boundary conditions for the classical linear dynamics of magnetization^34^. This profound and accurate prediction also shows the inverse proportionality of the frequency difference and the results are consistent with our experimental and numerical data.

## Discussion

In conclusion, using a versatile light-scattering technique, we have observed the IDM interaction in the inversion symmetry broken systems. The quantitative magnetic layer thickness-dependent measurements and careful analysis show the inverse proportionality of the frequency differences and confirm that the IDM interaction is a pure interfacial effect with maximum energy density of 1.2 mJ m^−2^ for Co with Pt underlayer. Furthermore, two different measurement methods, the magnetic field dependence and SW wavevector dependence, allow us to obtain identical results. These findings take us a step closer to boosting the IDM interaction leading to (meta-) stable skyrmion states for future data and memory devices. Finally, our numerical calculations confirm the asymmetric SW dispersion relations due to the IDM interaction and the inverse proportionality.

## Methods

### Thin film deposition

The sample of Pt(4 nm)/Co(0–2 nm)/AlO_x_(2 nm) and Pt(4 nm)/Co_48_Fe_32_B_20_(0–2 nm)/AlO_x_(2 nm) were prepared on Si/SiO_2_ substrates using DC magnetron sputtering with a base pressure of ∼7 × 10^−8^ mbar. To investigate the thickness dependence of IDM interaction, the ferromagnetic layers were grown in a wedge shape over 2 cm wide wafers with the help of an *in situ* moving shadow mask. AlO_x_ layer was obtained from plasma oxidation of 2-nm-thick Al layer as deposited on top of the ferromagnetic layers. The plasma oxidation process was carried out for 10 min in an *in situ* isolated chamber with a 0.1 mbar background pressure of oxygen and a power of 15 W.

### Brillouin light scattering

The samples are pasted on an angle controlled sample holder for the BLS measurement. The BLS spectra are measured by using a (3+3) pass tandem Fabry–Perot interferometer and a *p*-polarized (300 mW power and 532 nm wavelength) single longitudinal mode LASER is used as a light source. The DC external magnetic field is applied parallel to the film surface and perpendicular to the scattering plane. The back-scattered light from the sample is focused and collected. The *s*-polarized light is passed through the interferometer and the photomultiplier tubes^35^. All measurements are performed at room temperature. We use the applied magnetic field (0.01–1.18 T) and incident angle of light (25°–60°) corresponding to *k*_*x*_=0.0099–0.0205, nm^−1^ for magnetic field dependence and dispersion relation measurements, respectively. The accumulation time for each spectrum was about 60 min.

## Additional information

**How to cite this article:** Cho, J. *et al*. Thickness dependence of the interfacial Dzyaloshinskii–Moriya interaction in inversion symmetry broken systems. *Nat. Commun.* 6:7635 doi: 10.1038/ncomms8635 (2015).

## Supplementary Material

Supplementary Information Supplementary Figures 1-5, Supplementary Notes 1-7 and Supplementary References.
