# Supplementary material for: Thickness dependence of the interfacial Dzyaloshinskii–Moriya interaction in inversion symmetry broken systems
Source: Nat Commun. 2015 Jul 8;6:7635. doi: 10.1038/ncomms8635 (PMC4510697; doi:10.1038/ncomms8635)
Supplement: Supplementary Information — Supplementary Figures 1-5, Supplementary Notes 1-7 and Supplementary References. [file ncomms8635-s1.pdf]

SUPPLEMENTARY FIGURES

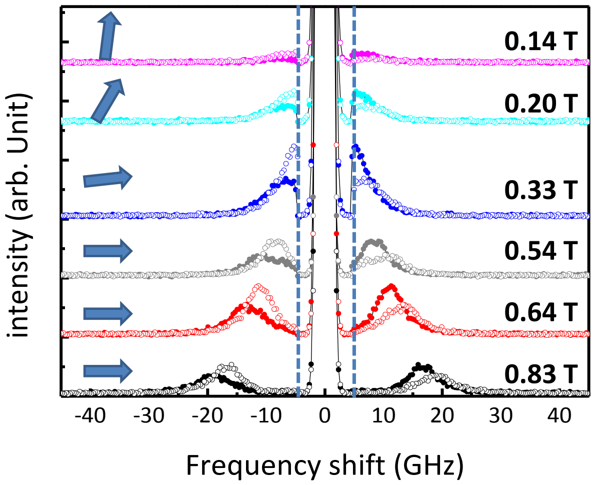

Supplementary Figure 1 | SW spectra with their mirror images for small in-plane field (0.14 ~ 0.83 T). When the in-plane magnetic field is larger than 0.5 T, the magnetization is out-of-plane, while it is smaller than 0.5 T, the magnetization is tilted. The schematic magnetization directions are shown as arrows in the left side of each spectrum. The dashed lines indicate Rayleigh scattering came from the interferometer shutter.

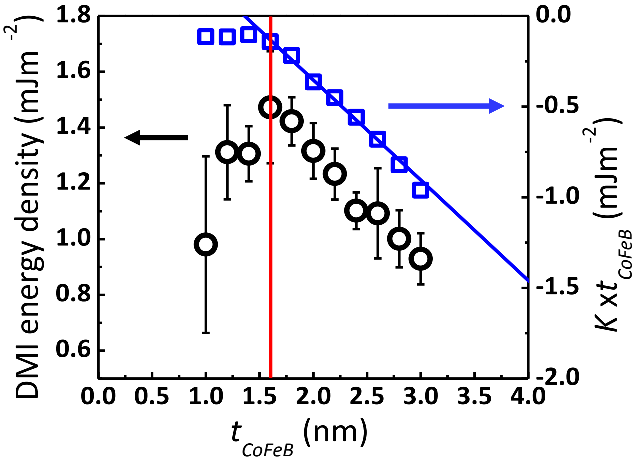

Supplementary Figure 2 | DM energy density and K_{eff} × t_{CoFeB} as a function of t_{CoFeB}. When t_{CoFeB} < 1.6 nm, the K_{eff} × t_{CoFeB} starts to deviate from the linear behaviour, and DM interaction shows the same behaviour.

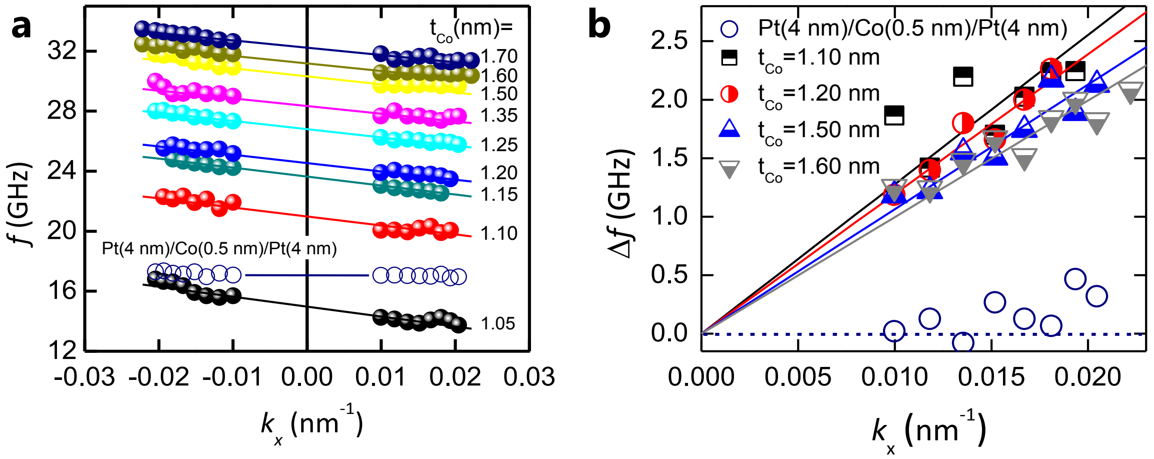

Supplementary Figure 3 | The SW dispersion relation and the linearity of the frequency differences for different Co thicknesses. a, The asymmetric dispersion relation measured by the BLS for various Co thicknesses. The open navy circles indicate the dispersion relation for a symmetric Pt (4 nm)/Co (0.5 nm)/Pt (4 nm) sample. For these measurements, the applied magnetic field is fixed at H_{ext} = 0.915 T. **b**, All Δf and linear fitting lines are visualized in one graph. The open navy circles show the Δf for the symmetric Pt (4 nm)/Co (0.5 nm)/Pt (4 nm) sample.

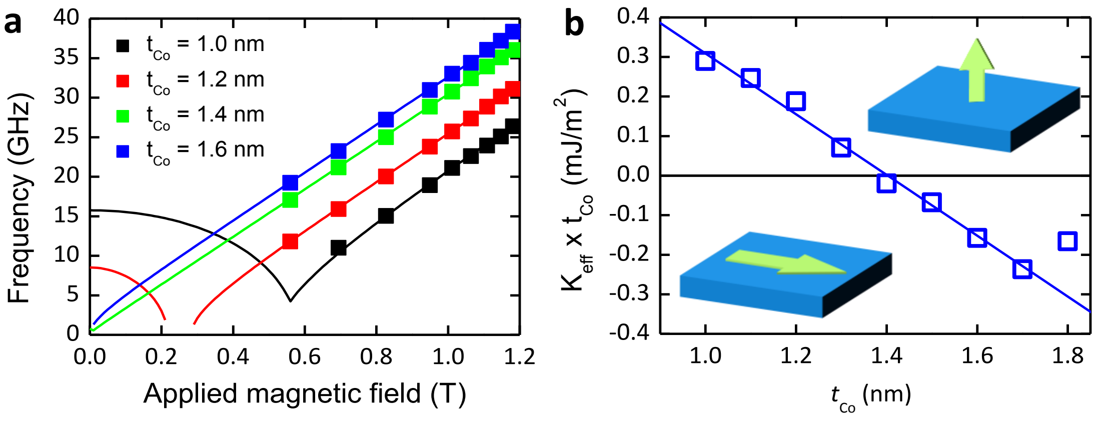

Supplementary Figure 4 | Measured SW frequencies and PMA energy densities using BLS. a, The field dependence SW resonance frequencies measured by BLS for various Co thickness. The squares indicate the median of the Stokes and anti-Stokes peak frequency and the solid lines are fits to Supplementary Eq. (3). **b**, $K_{eff} \times t_{Co}$ vs t_{Co} plot with linear fit. We extract K_s and M_s from the slope and *y*-axis crossing. Above $t_{Co} > 1.4$ nm, the effective anisotropy becomes negative and the easy axis of the sample is in-plane.

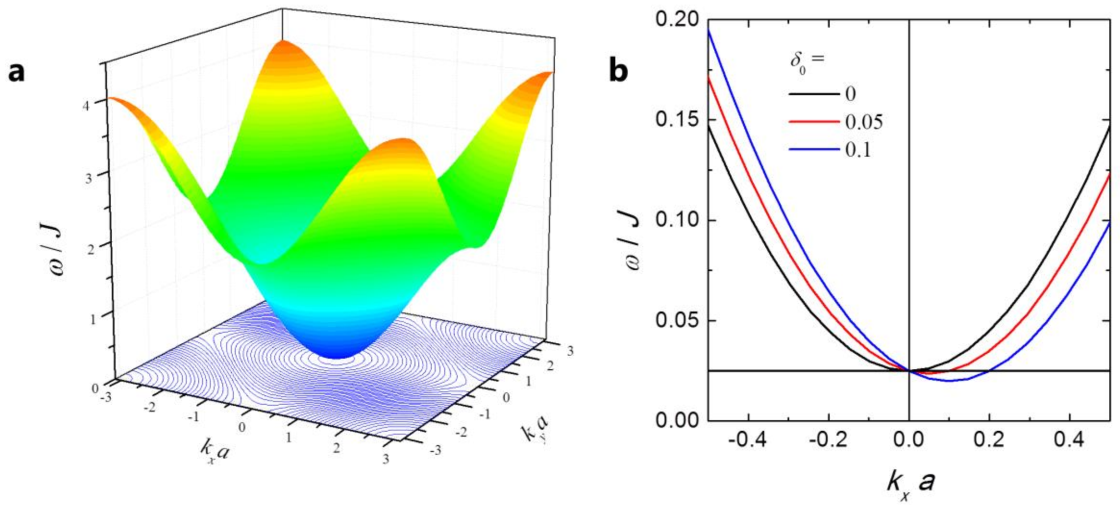

Supplementary Figure 5 | Calculated SW dispersion relations including the IDM interaction. **a**, Three dimensional SW dispersion relations for $k_x a$ and $k_y a$ without IDM interaction **b**, SW dispersion relations as a function of $k_x a$ with k_y=0 for δ_0=0, 0.05, 0.1.

SUPPLEMENTARY NOTES

Supplementary Note 1

Brillouin light scattering (BLS) technique and interfacial Dzyaloshinskii-Moriya (IDM) interaction

In order to determine the interfacial Dzyaloshinskii-Moriya (IDM) interaction, we measure the frequency difference (Δf) as a function of magnetic field or wavevector of the propagating spin wave (SW) by performing Brillouin light scattering (BLS).

All BLS data are governed by the so-called Damon-Eshbach (DE) mode including the contribution of the IDM interaction[1]:

$$f_{DE} = f_0\left(M_S, H_{ext}, K_U, A_{ex}, k_x\hat{\mathbf{x}}\right) + p\frac{\gamma D}{\pi M_S}k_x, \qquad (1)$$

where, f_0 is the SW frequency without the IDM contribution, H_{ext}, K_u, A_{ex}, p and $k_x = \frac{4\pi}{\lambda}\sin\theta$ are the external magnetic field, the magnetic anisotropy, the exchange stiffness, the polarity of the magnetic field ($p = \pm 1$) and the wave vector of the incoming light where θ is the incident angle of the light, respectively.

For the case of the field dependence, only the f_0 term is varied in Supplementary Eq. (1), and Δf does not vary. To check Supplementary Eq. (1) and reduce the uncertainty in the derived IDM energy density, we measure Δf as a function of magnetic field. The observed constant Δf as a function of the applied field validates the use of Supplementary Eq. (1) and allows us to accurately determine Δf, and hence, the derived IDM energy density.

During the measurements where we vary the *k*-vector, we fix the applied magnetic field at 0.915 T. As shown in Fig. 3b in the main text and as expected from Supplementary Eq. (1), Δf varies linearly with the *k*-vector as described by:

$$\Delta f = \left| f_{DE}\left(+k_x\hat{\mathbf{x}}\right) - f_{DE}\left(-k_x\hat{\mathbf{x}}\right)\right| = \frac{2\gamma D}{\pi M_S}k_x, \qquad (2)$$

where γ and D are the gyromagnetic ratio and the IDM energy density, respectively. By linear fitting, we directly extract the IDM energy. We conclude that both magnetic field and *k*-vector dependent measurements are well described by Supplementary Eq. (1) and (2).

Supplementary Note 2

Advantages of BLS to determine the IDM interaction energy density

Many different techniques are currently employed to study the IDM interaction, such as ferromagnetic resonance (FMR)[2], domain wall motion[3,4], and spin-polarized electron energy loss spectroscopy (SPEELS)[5]. However, FMR has not resulted in conclusive results as yet, and its ill-defined wavevector requires careful data analysis. Domain wall (DW) motion and nucleation based techniques are intrinsically complex as many aspects need to be taken into account e.g. DW energy profiles, pinning potential, etc. SPEELS can measure SW dispersion relations, however, the range of measurable wavevectors is 2 ~ 10 nm^{-1} (corresponding to 0.1 ~ 0.5 nm length scale) is too limited to draw conclusions. Furthermore, SPEELS cannot measure the field dependence which is required to confirm the source of the Δf. Furthermore, it requires alternative means to measure the saturation magnetization required to extract the IDM energy density.

In contrast to the above methods, BLS uses a well-defined SW wavevector, which is determined by the wavelength and incident/scattering angle of the laser beam. Moreover, BLS is able to detect propagating SW excitations with $\pm k$-wavevectors simultaneously (Stokes and anti-Stokes peaks)[6,7,8]. The range of usable wavevectors is 0.01 ~ 0.02 nm^{-1} corresponding to a length scale of 50 ~ 100 nm which is close to the length scale of skyrmions[9,10] with GHz range SW excitation. Utilizing the magnetic field and k-vector dependent BLS measurements the IDM energy density can be determined from the frequency difference between SWs with opposite ($\pm k$) wavevector. Furthermore, BLS allows for a direct measurement of the saturation magnetization (see Supplementary Note 6) and for local probing of samples as a small laser spot size is used. Specifically, we can perform local BLS measurements on ultrathin wedge shaped Pt/Co/AlO_x and Pt/CoFeB/AlO_x samples in order to investigate the thickness dependence of the IDM energy density as function of the Co (CoFeB) layer.

Supplementary Note 3

Asymmetric SW Dispersion for Out-Of-Plane Magnetization Geometry

Asymmetric SW dispersion is a finger print of the IDM interaction when the magnetization is in-plane configuration. Our experimental conditions are satisfied this conditions. However, Cortés-Ortuño *et al.*[11] pointed out the asymmetry vanishes when the magnetization is out-of-plane. Therefore, it must be examined in our experiments by reducing the in-plane applied field. BLS SW spectra with various in-plane magnetic fields of the 1.2-nm-thick Co sample are shown in Supplementary Fig. 1. In this figure, the largest peak which occurs around 0 GHz is due to elastically scattered light, so-called “Rayleigh scattering”, which is not related with magnetic signals. The peaks around 15 ~ 20 GHz with 0.83 T are typical BLS signals from the Pt/Co/AlO_x sample. The closed circles are measured spectra and the open circles are mirror spectra in order to show clearly the frequency differences. We only show rather large fields (> 0.5 T) spectra in the manuscript due to the measurement limitation of our BLS system. The vertical dashed lines which indicate near Rayleigh scattering came from the interferometer shutter, are unavoidable. For the case of H_{ext} < 0.33 T in our data, the spin orientations are changed from in-plane to out-of-plane (the blue arrows schematically indicates the magnetization directions). When the applied magnetic fields are less than 0.33 T, the peak position cannot be determined correctly, because of the shutter. Moreover, when the applied magnetic field is 0.14 T, the SW intensity is too small to confirm the correct peak positions. Therefore, unfortunately, we are not able to obtain meaningful spectra for fields smaller than 0.5 T, and this is the reason why we only show spectra for rather large fields where the magnetization direction is in-plane. Because of the limitation of our measurement system, we cannot determine whether the asymmetric dispersion is vanished for the out-of-plane magnetization or not.

Supplementary Note 4

The non-linear behaviour of frequency difference of Pt/CoFeB/AlO_x

In Fig. 2b in the main text, the frequency difference of Pt/CoFeB/AlO_x shows a maximum value at 1.6 nm, while Pt/Co/AlO_x shows clear linear behaviour. Physical reason of such non-linear behavior of Pt/CoFeB/AlO_x must be addressed. In order to resolve the un-expected behavior of Pt/CoFeB/AlO_x, we plot together $K_{eff} \times t_{\mathrm{CoFeB}}$ and DM energy density via t_{CoFeB} (thickness of CoFeB) in Supplementary Fig. 2. It is clear that the linear behaviour is broken in the $K_{eff} \times t_{\mathrm{CoFeB}}$ vs. t_{CoFeB} plot, when $t_{\mathrm{CoFeB}} < 1.6$ nm. Based on our observation, we speculate the interface quality is changed due to the too thin ferromagnetic layer. Such deviation is usually observed in $K_{eff} \times t$ vs. t plots for PMA materials (see Supplementary Note 6). The onset of the non-linear behaviour in the frequency difference or DM energy density is exactly the same thickness. Therefore, it implies that the non-linear behaviour in Fig. 2b in the main text is due to the degradation of the interface quality.

Supplementary Note 5

The SW *k*-vector dependent BLS measurements for a symmetric sample.

In this section, we discuss the BLS measurements for nominal symmetric-interface samples such as Pt (4 nm)/Co (0.6 nm)/Pt (4 nm). As described in the main text, from this nominally symmetric structure we expect negligible or zero IDM interaction. SW *k*-vector dependent measurements are performed similarly as used for Fig. 4 of the main text and are shown in Supplementary Fig. 3. Open navy circles in Supplementary Fig. 3a indicate the SW dispersion relation. Due to the limited *k*-vector range, we only observe the symmetric dispersion, which implies a small IDM interaction. Supplementary Fig. 3b shows the correlation ($\Delta f = \left| f_{DE}\left(+k_x\hat{\mathbf{x}}\right) - f_{DE}\left(-k_x\hat{\mathbf{x}}\right) \right| = \frac{2\gamma D}{\pi M_S} k_x$) between the frequency differences and SW *k*-vector. No significant IDM interaction is observed by using BLS. To elucidate, two reasons are suggested; first, our examined system is more symmetric compared to the other reports (Refs. 8 and 13 in main text), and second, the IDM interaction might be small and cannot be detected by BLS as a small Δf falls within the detection limit. Therefore, a small frequency, which indicates a small or negligible IDM energy density *cannot* be identified by BLS.

For the BLS measurements, a tandem interferometer with a free spectral range (FSR) of 75 GHz and a 2^8 multichannel analyser is used. The frequency resolution in the measured Stokes and anti-Stokes peaks in the BLS spectra can be determined by using FSR/2^8 GHz. Therefore, the frequency resolution of the BLS setup is approximately 0.29 GHz. Since the correlation between the frequency difference and the IDM energy density is given by $\Delta f = \frac{2\gamma D}{\pi M_S} k_x$, we can simply deduce that the resolution of the obtained IDM energy density is about D = 0.164 mJm^{-2} with a saturation magnetization of M_s = 1100 kAm^{-1} , γ = 2.37×10^{11} T^{-1}s^{-1}, k_x = 0.0167 nm^{-1}, and Δf = 0.29 GHz, respectively.

Supplementary Note 6

Determination of the saturation magnetization and anisotropy energies

In this section, we demonstrate the SW dispersion relation without the IDM interaction. First, in order to define the SW frequency without the IDM interaction, the median value of the Stokes and anti-Stokes peak are taken to determine the perpendicular magnetic anisotropy energy and the saturation magnetization. As illustrated in Supplementary Fig. 4a, the applied magnetic field dependence of SW are measured by BLS for various Co thicknesses (t_{Co} = 1.0, 1.2, 1.4, and 1.6 nm). Since the applied magnetic field is perpendicular to the magnetization creating the surface SW mode, the SW excitation frequencies are given by[12]:

$$f_0 = \frac{\gamma}{2\pi}\sqrt{\left[H_{ext}\cos\theta - \left(4\pi M_s - \frac{2K_u}{M_s}\right)\sin^2\theta\right]\left[H_{ext}\cos\theta - \left(4\pi M_S - \frac{2K_u}{M_s}\right)(\cos^2\theta - \sin^2\theta)\right]}$$

(3)

where, γ is the gyromagnetic ratio (= 2.37×10^{11} $T^{-1}s^{-1}$), θ is the angle between the magnetization and the sample plane, K_u is the perpendicular uniaxial anisotropy constant, H_{ext} is the external magnetic field, M_s is the saturation magnetization, respectively. In this equation, the contributions of dipolar field and exchange energy have been neglected as is justified in the ultrathin limit. Consequently, the measured SW frequencies and the fitted curves show a good correspondence as shown in Supplementary Fig. 4a. For the case of t_{Co} > 1.4 nm, the frequencies of the propagating SWs differ from the thinner thicknesses, which means that the effective uniaxial anisotropy ($K_{eff} = 2K_s/t\text{-}1/2\mu_0 M_s^2$) is changed from positive (out-of-plane) to negative (in-plane) values. To elaborate, we plot the anisotropy energy density ($K_{eff}\times t_{Co}$) as a function of t_{Co} in Supplementary Fig. 4b. From this plot, we determine the slope and y-crossing, corresponding to the volume anisotropy ($-1/2\mu_0 M_s^2$) and the surface anisotropy (K_s)[13], respectively. This allows us to extract M_s directly from the BLS measurements. M_s is the only necessary physical quantity to convert the measured Δf to the IDM energy density. The obtained K_s is 0.54 mJm^{-2} and M_s is 1100 kAm^{-1}, which is about 78.5% of the bulk Co value.

Supplementary Note 7

Double time Green's function for SW dispersion relations

To calculate the SW dispersion relation in ultrathin ferromagnetic layers and super-lattices, the double time Green′s function method is widely used[14,15,16,17,18]. It is a well-developed method in statistical physics[19] and magnetism[20].

We now briefly review the double time Green′s function to obtain the SW dispersion relations of *N* atomic ferromagnetic layers with DM interaction. The Hamiltonian with DM interaction in terms of the spin operator is given by[21, 22]:

$$H = -g\mu_B H_{ext}\sum_{i} S_i^z - \sum_{\langle i,j\rangle} J_{ij}\mathbf{S_i}\cdot\mathbf{S_j} - K_u\sum_{\langle i,j\rangle} S_i^z S_j^z - K_s\sum_{\langle i,j\rangle'} S_i^z S_j^z + \sum_{\langle i,j\rangle''} D_{ij}\hat{\mathbf{z}}\cdot\left(\mathbf{S_i}\times\mathbf{S_j}\right), \quad (4)$$

where, J_{ij} and D_{ij} are the isotropic inter-atomic Heisenberg and DM exchange energies between the *i*-th and *j*-th spins, and K_u and K_s are the bulk and surface uniaxial anisotropy energies. $\langle i,j\rangle, \langle i,j\rangle', \langle i,j\rangle''$ denote the summations of the nearest neighbours. The last DM interaction term can be rewritten as

$$H_{DMI} = \sum_{\langle i,j\rangle''} D_{ij}\left(S_i^x S_j^y - S_i^y S_j^x\right). \quad (5)$$

Following Ref. 16, the double time Green function can be defined as:

$$G_{ij}\left(t,t'\right) = \left\langle\left\langle b_i^+\left(t\right) \mid b_j^-\left(t'\right)\right\rangle\right\rangle. \quad (6)$$

The equation of motion for G_{ij} is

$$i\frac{dG_{ij}\left(t,t'\right)}{dt} = \left\langle\left[b_i^+\left(t\right), b_j^-\left(t'\right)\right]\right\rangle\delta\left(t-t'\right) - \left\langle\left\langle\left[H, b_i^+\left(t\right)\right] \mid b_j^-\left(t'\right)\right\rangle\right\rangle, \quad (7)$$

and the higher order Green′s functions are decoupled by the random phase approximations, the set of differential equations for N-atomic ferromagnetic layers can be obtained[22].

We define the normalized energy quantities as $\delta_0 = D/J$, $k_u = K_u/J$, $k_{s1} = K_{s1}/J$, and $k_{sN} = K_N/J$. k_x, k_y, and a are the x and y component of the SW vector and the lattice constant, respectively. In these calculations, we assume a simple cubic lattice structure, but this model can be extended for *bcc* and *fcc* structures[22]. The DM interaction contribution is developed with the number operator, $\hat{n}_i = b_i^+ b_i^-$ [16]:

$$\sum_{i,j} D_{ij}\left(S_i^x S_j^y - S_i^y S_j^x\right) = \frac{1}{4}\sum_{i,j} 2iD_{ij}\left(b_i^- b_j^+ - b_i^+ b_j^-\right) \tag{8}$$

$$\cong D\sum_i \left(\sin\left(k_x a\right) + \sin\left(k_y a\right)\right)\hat{n}_i \,. \tag{9}$$

From the matrix equations, we obtain the series of the Green′s functions and eigenvalues of $\Delta(E)$ for a given k-vector. The N eigenvalues correspond to SW excitation energies, and corresponds with the SW dispersion relation. The typical SW excitations for the lowest energies are shown in Supplementary Fig. 5a and b for N = 2 simple cubic ferromagnetic layers with k_u = 0.01, k_{s1} = $k_s N$ = 0.01 and δ_0=0, 0.05, 0.1.

SUPPLEMENTARY REFERENCES

[1] Moon, J.-H., Seo, S.-M., Lee, K.-J., Kim, K.-W., Ryu, J., Lee, H.-W., McMicheal, R. D. and Stiles, M. D. Spin-wave propagation in the presence of interfacial Dzyaloshinskii-Moriya interaction. *Phys. Rev. B* **88**, 184404 (2013).

[2] Vlaminck, V. and Bailleul, M. Current-induced Spin-Wave Doppler Shift. *Science* **322**, 410-412 (2008).

[3] Emori, S., Bauer, U., Ahn, S.-M., Martinez, E. and Beach, G. S. D. Current-driven dynamics of chiral ferromagnetic domain walls. *Nature Mater*. **12**, 611-616 (2013).

[4] Pizzini, S. *et. al.,*Chirality-Inducd Asymmetric Magnetic Nucleation in Pt/Co/AlO_x Ultrathin Microstructures. *Phys. Rev. Lett.* **113**, 047203 (2014).

[5] Zakeri, Kh., Zhang, Y., Prokop, J., Chuang, T.-H., Sakr, N., Tang, W.X. and Kirschner, J. Asymmetric Spin-Wave Dispersion on Fe(110): Direct Evidence of the Dzyaloshinskii-Moria Interaction. *Phys. Rev. Lett.* **104**, 137203 (2010).

[6] Di, K., Zhang, V. L., Lim, H. S., Ng, S. C., Kuok, M. H., Yu, J., Yoon, J., Qiu, X., and Yang, H. Direct Observation of the Dzyaloshinskii-Moriya Interaction in a Pt/Co/Ni Film, *Phys. Rev. Lett.* **114**, 047201 (2015).

[7] Stashkevich, A. A., Belmeguenai, M., Roussigné, Y., Cherif, S.M., Kostylev, M., Gabor, M., Lacour, D., Tiusan, C., Hehn, M. Non-reciprocity of spin wave propagation induced by the interface Dzyaloshinskii-Moriya interaction in Py/Pt film structures, Preprint at http://arXiv.org/abs/1411.1684 (2014).

[8] Belmeguenai, M., Adam, J.-P., Roussigné, Y., Eimer, S., Devolder, T., Kim, J.-V., Cherif, S. M., Stashkevich, A., Thiaville, André, Interfacial Dzyaloshinskii-Moriya interaction in perpendicularly-magnetized Pt/Co/AlOx ultrathin films measured by Brillouin light spectroscopy, Preprint at http://arXiv.org/abs/1503.00372 (2015).

[9] Fert, A., Cros, V. and Sampaio, J. Skyrmions on the track. *Nature Nanotech*. **8**, 152-156 (2013).

[10] Rohart, S. and Thiaville, A. Skyrmion confinement in ultrathin film nanostructures in the presence of Dzyaloshinskii-Moriya interaction. *Phys. Rev. B.* **88**, 184422 (2013).

[11] Cortes-Ortuno, D. & Landeros, P. Influence of the Dzyaloshinskii-Moriya interaction on the spin-wave spectra of thin films. *J. Phys. Cond. Matter* **25**, 156001 (2013).

[12] Dutcher, J. R., Heinrich, B., Cochran, J. F., Steigerwald, D. A. and Egelhoff, Jr. W. F. Magnetic properties of sandwiches and superlattice of fcc Fe(001) grown on Cu(001) substrates. *J. Appl. Phys.* **63**, 3464-3466 (1988).

[13] Johnson, M. T., Bloemen, P. J. H., Broeder, F. J. A. and de Vries, J. J. Magnetic anisotropy in metallic multilayers. *Rep. Prog. Phys.* **59**, 1409-1458 (1996).

[14] Selzer, S. and Majlis, N. Theory of the surface magnetization profile and the low-energy, spin-polarized, inelastic celctron scattering off insulating ferromagnets at finite T. *Phys. Rev. B* **26**, 404-414 (1982).

[15] Endo, Y. and Ayukawa, T. Magnetic properties of Heisenberg-type ferromagnetic films with a sandwich structure. *Phys. Rev. B* **41**, 6777–6782 (1990).

[16] Haubenreisser, W., Brodkorb, W., Corciovei, A., and Costache, G. A status report of Green’s function theory in uniaxial ferromagnetic unpinned thin films. *Phys. Stat. Sol. (b)* **53**, 9–40 (1972).

[17] Bruno, P. Theory of the Curie temperature of Cobalt-based ferromagnetic ultrathin film and multilayers. *J. Magn. Soc. Jpn*., **15**, 15 (1991).

[18] Bruno, P. Spin-wave theory of two-dimensional ferromagnets in the presence of dipolar interactions and magnetocrystalline anisotropy. *Phys. Rev. B* **43**, 6015-6021 (1991).

[19] Zubarev, D. N. Double-time Green functions in statistical physics. *Sov. Phys. USP.* **3**, 320-345 (1960).

[20] Tyablikov, S. V., *Methods in the quantum theory of magnetism*, Plenum, (1967).

[21] You, C.-Y. Curie temperature of ultrathin ferromagnetic layer with Dzyaloshinskii-Morita interaction. *J. Appl. Phys.* **116**, 053902 (2014).

[22] Diep, H.-T., Levy, J. C. S., and Nagai, O. Effects of surface spin waves and surface anisotropy in magnetic thin film at finite temperatures. *Phys. Stat. Sol. (b)* **93**, 351-361 (1979).
